# Software Assurance Using Structured Assurance Case Models

**DOI:** 10.6028/jres.115.013

**Published:** 2010-06-01

**Authors:** Thomas Rhodes, Frederick Boland, Elizabeth Fong, Michael Kass

**Affiliations:** Software and Systems Division, Information Technology Laboratory, National Institute of Standards and Technology, Gaithersburg, MD 20899-8970

**Keywords:** product assurance, software assurance, software assurance case, software security, structured assurance case model, structured software assurance model

## Abstract

Software assurance is an important part of the software development process to reduce risks and ensure that the software is dependable and trustworthy. Software defects and weaknesses can often lead to software errors and failures and to exploitation by malicious users. Testing, certification and accreditation have been traditionally used in the software assurance process to attempt to improve software trustworthiness.

In this paper, we examine a methodology known as a structured assurance model, which has been widely used for assuring system safety, for its potential application to software assurance. We describe the structured assurance model and examine its application and use for software assurance. We identify strengths and weaknesses of this approach and suggest areas for further investigation and testing.

## 1. Introduction

Software assurance is an important part of the software development process to reduce risks and ensure that the software is trustworthy. The critical importance of establishing and assuring dependability and trustworthiness (e.g., safety, security, reliability, etc.) of systems and/or software in avionics, industrial control systems and other safety and mission-critical systems has long been recognized.^1^ The key purpose of a software assurance assessment is to show that the system, as designed and built, functions as intended (functional and dependable) and is free from defects and vulnerabilities that might be introduced intentionally or unintentionally. Inspection, testing, certification and accreditation, and configuration management have all been traditionally used in the software assurance process with mixed results. In the report, “*Software for Dependable Systems—Sufficient Evidence*?” by the Committee on Certifiably Dependable Software Systems of the National Research Council, [1] the Committee recommends a strong *evidence-based approach* for assessing and assuring dependability in software systems that argues for and justifies dependability claims based on explicit evidence supporting such arguments and claims.

An evidence-based approach to software system assurance can be made through use of an assurance case methodology based on a structured assurance case model. Structured assurance case models have been used widely in the United Kingdom [2, 3] and United States [4] for developing safe and secure systems. Proponents of this approach have argued that a structured assurance case model provides a common framework for bringing together best practices in the safety, security, and reliability domains to achieve a single, unified assurance case [5]. However, the effectiveness of the structured assurance case model as a mechanism for assuring software system properties, such as security, reliability, availability, and others, remains a subject of continuing investigations. Currently, various on-going research activities are investigating application of the assurance case model approach for assuring software properties, such as, safety, security, and reliability [5, 6, 7].

In this paper, we describe the structured assurance case model and examine its application to software assurance with a simple software-based system example.

## 2. Software Assurance

Various definitions of software assurance have been given. For example, Wikipedia [8] references several definitions of software assurance on their software assurance web page. Many bear resemblance to the definition of software assurance provided in the IEEE Standard Glossary of Software Engineering Terminology (IEEE 610.12) [9]. The National Aeronautics and Space Administration (NASA) Goddard Spaceflight Center has adopted the IEEE definition. Other definitions, such as that of the National Institute of Standards and Technology (NIST) Software Assurance Metrics and Tool Evaluation (SAMATE) project [10], and the Department of Homeland Security (DHS) Software Assurance (SwA) program [11] have adopted the IEEE definition with some extensions relating to software trustworthiness and security, as described below:

- NIST/SAMATE project:
  “The planned and systematic set of activities that ensures that software processes and products conform to requirements, standards and procedures in order to help achieve:
  – Trustworthiness—no exploitable vulnerabilities exist either of malicious or unintended origin, and
  – Predictable execution—justifiable confidence that software, when executed, functions as intended.”
- DHS SwA Program: “Software assurance (SwA) is the level of confidence that software is free from vulnerabilities, either intentionally designed into the software or accidentally inserted at any time during its life cycle, and that the software functions in the intended manner.” [12].

## 3. Structured Assurance Case

A structured assurance case is “*a documented body of evidence that provides a convincing and valid argument that a specified set of critical claims regarding a system ’s properties are adequately justified for a given application in a given environment* [13].” Much like a legal case presented in a courtroom, an assurance case is a comprehensive presentation of evidence and arguments that support claims about properties or behaviors of a product or system, such as, security, safety reliability, etc. Hence, the structured assurance case provides a structured, composable, and traceable model for demonstrating and verifying the plausibility and strength of claims made about a property of the product or system in question.

### 3.1 Structured Assurance Case Model

**Fig. 1:**
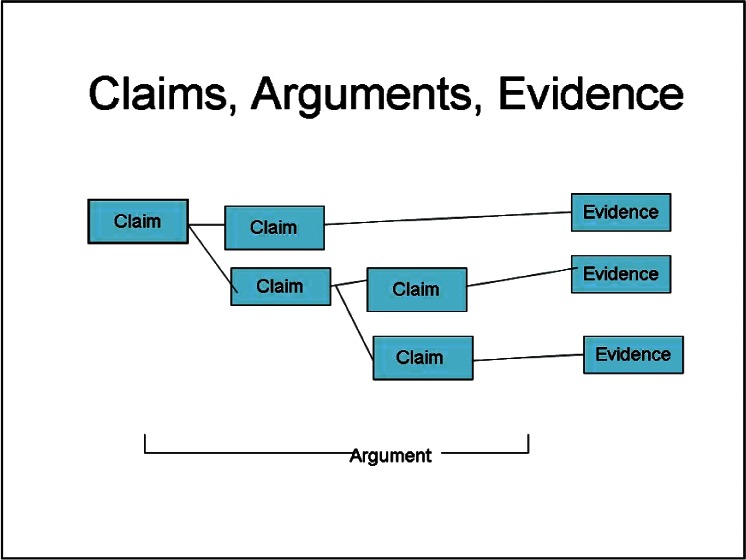
Representation of a Structured Assurance Case Model.

The structured assurance case model is represented as a directed graph whose nodes consist of claims, arguments, and evidence elements. Figure 1 is a graphical representation of an assurance case, showing the relation between claims, arguments, and evidence. The left-most claim is the top-level, overall claim. It is decomposed and supported by two sub-claims. The argument is that if all sub-claims are valid then the overall claim is valid. The argument continues with further sub-claims until, ultimately, a claim or sub-claim is supported by evidence that is sufficient, objective, and reproducible. This representation of an assurance case model [2] has explicit elements for “claims” and “evidence” with the argument being implied from the structure and logical relationships among the claims and evidence elements. Other notations, however, of a structured assurance case model include an explicit element for the “argument” along with claims and evidence and in some cases provides notations for assumptions and conditions.

Currently, the Object Management Group (OMG) System Assurance Platform Task Force (PTF) (formerly the Software Assurance Special Interest Group) is including the structured assurance case model as part of a common framework of standards for analysis and exchange of information related to systems assurance and trustworthinesss [14]. This Framework and Metamodel will provide a machine-readable repository for assurance case artifacts, such as, claims, arguments, and evidence, and enable software development and testing tools to exchange and share information across the software lifecycle in support of software assurance.

The standards group, ISO/IEC JTC1 Sub-Committee 7 WG7, is also including the structured assurance model as a revision to Part 2 of the ISO/IEC 15026, Systems and Software Assurance standard [15].

### 3.2 Claims and Sub-Claims

A claim is a statement asserting some characteristic, property, or behavior of the software or system that can be evaluated for truthfulness, is demonstrable, and is supported by arguments based on objective evidence. A claim may be further decomposed into sub-claims, and expressed either as a positive or negative statement.

For example, one can declare positive claims about the requirements-based, quality properties of software, such as its dependability or availability, or one can make negative claims about the same software by claiming that the code does not contain specific weaknesses and vulnerabilities in the design and implementation that could be exploited to break or compromise the system.

### 3.3 Arguments

Arguments are logical propositions intended to support a claim through reasoning or logic that links evidence to a claim. Arguments define the relationships directly linking each claim and/or sub-claims, and piece of evidence, used by an argument to the claims immediately supported by the argument. An argument is the explanation of how the evidence can be interpreted as supporting a claim or sub-claim.

Arguments can also include any unusual events or conditions that are within the context of the claim. The argument can contain considerations of potential causes of failure and appropriate corrective actions if failure occurs. Hence, an argument may include conditions, assumptions, and judgments about the system, its use, and its operational environment, threats, and likelihood of occurrence, for which the claims and evidence are being marshaled as part of an overall assurance case.

### 3.4 Evidence

Evidence is information used to support a claim. Ideally, evidence should be objective, reproducible, repeatable, and non-disputable. Evidence is key to making a credible assurance case. Without evidence, there is no way to substantiate the claim.

The sources of evidence will depend in part on the availability of artifacts. The evidence data collection may be conducted formally, informally or semiformally.

Evidence comes in many different forms, so it is impossible to dictate what kind of evidence or argument is appropriate for every situation. Evidence may be in the form of an artifact which could be automatically, semi-automatically or manually produced and demonstrated. Evidence must be traceable to its source and method of origination. The evidence may consist of test results, formal analyses, simulation results, hazard analyses, modeling, inspections, and can include deterministic, probabilistic, and qualitative data or information [14]. Examples of evidence data might be software artifacts, methodologies, development processes, testing results, people or programmer expertise and experience credentials, development environments, operational environments, or regulatory compliance.

## 4. Composing a Structured Software Assurance Case Model

The structured assurance case model defined previously has potential in providing a framework for an effective software assurance case. The model may be developed in a top-down approach, bottom-up approach, or a mix of both top-down and bottom-up approaches.

The basic steps in implementing a structured assurance case are to:

– Define or assert a top level claim about a software or system property which is to be shown.
– Consider decomposing the top level claim into smaller related sub-claims.
– Identify or provide the supporting evidence for the sub-claims.
– Develop a set of arguments that link claims/subclaims to evidence to support the claims/subclaims.
– State any assumptions, judgments, and conditions underlying the claims, arguments, and evidence.
– Evaluate the strength and sufficiency of the assurance case evidence and arguments in substantiating the claims and sub-claims.

The process is both cumulative and iterative. Assurance claims and sub-claims may be decomposed to any level of granularity until necessary and sufficient evidence is obtained in supporting the satisfaction of a claim or a sub-claim. The structured assurance case then rests upon the aggregation of all sub-claims and arguments, each supported by evidence, which collectively satisfies a top-level claim. Ideally, objective measures of whether the evidence is of high quality and sufficient are desirable. However, in practice, this may be difficult and *sufficiency* will often be decided by some combination of objective evidence (e.g., test results) and expert opinion that collectively provides strong and plausible evidence supporting an argument and claim.

### 4.1 Notations and Tools

Structuring assurance cases so they can be understood is a challenge. Due to the massive amount of evidence that may be needed to demonstrate an assurance case for moderately-sized software, and to improve human capability for reviewing and visualizing an assurance case, automated tools have been developed. Examples of notations for which tools have been developed, include:

– Goal Structuring Notation (GSN) [16]
– Claims-Arguments-Evidence (CAE) [17]

Both use a graphical notation for representing the structure of an assurance case. There are similarities and differences in notations among different tools. For example, the GSN notation defines node types for Goals (claims), Strategy (argument), and Solution (evidence), with supporting nodes that include Assumptions, Justifications, Context, Models, and Notes. CAE defines nodes for Claims, Arguments, and Evidence. GSN has a goal-oriented view that supports a top-down approach in developing the structured assurance case beginning with claims, while the CAE supports a bottom-up view that uses evidence to determine which claims can be made. Currently, within the Object Management Group (OMG) there is an effort to produce a standard that encompasses concepts from both of these notations [14].

### 4.2 Sources of Evidence

The sources of evidence will depend in part on the availability of artifacts. The evidence data collection may be conducted formally, informally or semiformally.

Data facts, as evidence, are collected to support the argument that the software will satisfy particular claims for software assurance.

### 4.3 A Simple Structured Assurance Case Example

As an illustration of a structured assurance case model approach for a software-based product, a simple example is shown for an automated teller machine (ATM). This assurance-related claim is derived from a presumed security specification requirement for an ATM that states that the ATM must not allow un-authorized access to a bank account.

**Fig. 2:**
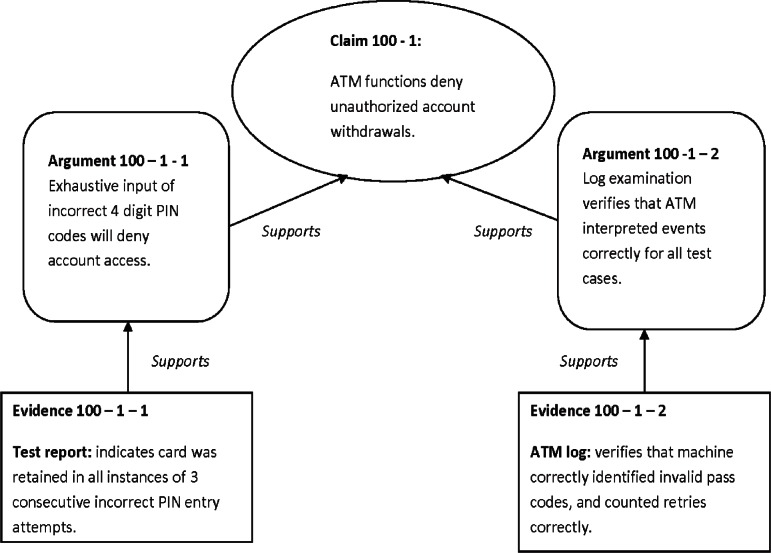
Assurance Case Model for ATM.

Figure 2 below illustrates this portion of the ATM example using a simple claims-arguments-evidence model. Note, that in practice, such a model would be a more realistic and comprehensive model of sub-claims, arguments and evidence.

## 5. Potential Benefits, Issues and Challenges

The structured assurance case model has been extensively used and shown to be an effective approach for assuring safety in avionics and other complex systems [2]. Other applications have demonstrated its use for assuring systems security [5, 6, 7] or as a framework for a unified approach to safety, security, and reliability. Work done by Ankrum and Kromholz of MITRE, illustrated the use of structured assurance cases for mapping and analyzing assurance standards, and for analyzing a practical security-critical system [13]. Associated graphical notations can provide a visible model for human use that is comprehensive, understandable, and which can provide traceability between the model elements of claims, arguments, and evidence.

The structured assurance case model, applied to software assurance, can support various stakeholder roles and needs, including those of the developer, acquirer, and certifier throughout the system life-cycle. The structured assurance case model provides a framework for identifying critical properties of a software system, such as, safety, security, and dependability, and ensuring that these are addressed during development, implementation, and testing. Furthermore, an assurance framework enables capturing lifecycle artifacts that provide the evidence needed to support claims about these requirements. The structure and hierarchy of the structured assurance case model can help identify gaps between claims, arguments, and evidence, and provide a consistent approach for software assurance.

The structured assurance case model offers an additional approach to software assurance that has traditionally been provided through certification and accreditation activities by providing traceability. Thus, use of this approach can improve the overall software assurance process.

However, the use of structured assurance case models for software assurance is an on-going topic of research and case-studies. There are still open issues surrounding the use of the structured assurance method for software assurance, including:

– Measuring the effectiveness of the structured assurance case model for software assurance.
– Determining what and how much evidence is sufficient for verifying a claim/sub-claim.
– Ensuring that the quality of evidence is satisfactory.
– Ensuring an appropriate level of detail or granularity of sub-claims.
– Ensuring that relationships among claims, arguments, and evidence are clear and explicit.
– Managing large, complex structured assurance case models.
– Improving guidance on how to efficiently gather, merge, and review arguments and evidence.
– Developing automated tools to analyze structured assurance cases.

Some ongoing issues with the structured assurance case model approach include:

- Difficulty in transforming existing safety and security requirements into the structure of an evidence-based assurance model. Standards for defining safety and security requirements for application domains often specify a structure and format that do not easily translate into a structured assurance case model. The result can be an assurance model that is incomplete, contradictory, and not aligned with the requirements.
- Existing assurance models in safety and security rely heavily on evidence of compliance to standards for lower levels of assurance (e.g., safety integrity levels (SIL) and evaluation assurance levels (EAL) respectively), with the assumption that adherence to those standards validates the overall assurance claim. Use of an evidence-based model can facilitate the use of artifacts generated by tools as evidence against the actual system itself, providing a stronger claim of safety, security or other property for lower levels of assurance.
- Assurance modeling of “system of systems” adds another layer of complexity to the assurance case. While a system may be deemed safe or secure by itself in a particular environment, the introduction of other systems into that environment increases the complexity of the assurance model and must be considered and evaluated as part of a larger system. Assurance case models today do not address the assurance of systems of systems.

## 6. Conclusions

Use of a structured assurance case method shows promise for use in assurance of software properties, such as, safety, security, reliability, and others. This model provides an organized, structured approach to software assurance based on claims, arguments and evidence, and provides a means of traceability among these elements.

The model appears useful throughout the software development lifecycle by providing a framework where intended product claims can be identified early in the development cycle and used to identify system requirements upon which these claims can be based, and for which arguments and evidence can be established during development to support these claims.

However, further work is needed in developing models for different software system properties and examining relationships and patterns that may exist within and among these models. Further work is also needed to develop automated methods to handle and process potentially large and complex assurance models, and support definition, maintenance, and revision of large assurance models, amounts of evidence, and to develop methods for objective measurements for evaluating the “*quality*” of the model in providing a strong software system assurance case.

## Footnotes

1 Certain commercial equipment, instruments, or materials are identified in this paper to foster understanding. Such identification does not imply recommendation or endorsement by the National Institute of Standards and Technology, nor does it imply that the materials or equipment identified are necessarily the best available for the purpose.
